# Transcranial direct current stimulation (tDCS) eliminates the other-race effect (ORE) indexed by the face inversion effect for own versus other-race faces

**DOI:** 10.1038/s41598-022-17294-w

**Published:** 2022-07-28

**Authors:** Ciro Civile, I. P. L. McLaren

**Affiliations:** School of Psychology, College of Life and Environmental Sciences, University of Exeter, Exeter, UK

**Keywords:** Psychology, Human behaviour

## Abstract

We investigate here individuals’ reduced ability to recognise faces from other racial backgrounds, a robust phenomenon named the other-race effect (ORE). In this literature the term “race” is used to refer to visually distinct ethnic groups. In our study, we will refer to two of such groups: Western Caucasian (also known as White European) and East Asian e.g., Chinese, Japanese, Korean. This study applied the tDCS procedure (double-blind, 10 min duration, 1.5 mA intensity, targeting Fp3 location), developed in the perceptual learning literature, specifically used to remove the expertise component of the face inversion effect (FIE), which consists of higher recognition performance for upright than inverted faces. In the tDCS-sham condition (N = 48) we find a robust ORE i.e., significantly larger FIE for own versus other-race faces due to higher performance for upright own-race faces. Critically, in the anodal-tDCS condition (N = 48) the FIE for own-race faces was significantly reduced compared to sham due to impaired performance for upright faces thus eliminating the cross-race interaction index of the ORE. Our results support the major role that perceptual expertise, manifesting through perceptual learning, has in determining the ORE indexed by the FIE.

## Introduction

The race of a face is often one of its most salient features. We can rapidly categorise two faces that belong to two different racial groups as, for example, Western Caucasian versus East Asian, by means of shape and superficial colour information^1^. More importantly, within the racial group with which we are most familiar, we are highly accurate at recognising previously seen faces^2^. However, one of the most robust and well documented face recognition phenomena is our impaired ability in recognising faces from other races^3,4^. This has been named the Other-Race Effect (ORE) and is one of the best-replicated phenomena in the face recognition literature as established by several reviews and meta-analyses that have considered 30 years of research on the ORE^5–7^.

In the lab, the ORE is typically demonstrated through a standard recognition paradigm, in which subjects are asked to recognise faces previously seen during a study phase, intermixed with novel faces. The ORE is then indexed by a cross-over interaction between the race of subjects and the race of faces in recognition accuracy^8,9^. A long-standing debate regarding the nature of the ORE is whether it can be used as a measure of racial bias, or instead is a face recognition phenomenon caused by reduced perceptual expertise that we have for other-race faces. However, some authors have also proposed a hybrid approach where both perceptual expertise mechanisms and social factors play a key role in determining the ORE. In what follows, we give the overall background to this literature, and then introduce our contribution, which is to deploy a neurostimulation technique that significantly reduces (perhaps even eliminates) perceptual expertise. The basic idea of this study is that if the ORE is substantially due to perceptual expertise giving an advantage to own-race faces, then the effect should disappear under our neurostimulation manipulation. This would provide direct evidence of the perceptual expertise mechanisms involved in the ORE and advance our understanding of this phenomenon.

## Background

In early studies, social scientists had interpreted this phenomenon as an indication of how observers, particularly those with more prejudiced racial attitudes, would not be motivated to differentiate members of other races, which would then lead to a weaker memory for other-race faces^10^. Throughout the years, various versions of this motivational account linked with social categorisation have been proposed for the ORE^11,12^. But, perhaps, the most developed explanation based on social and motivational factors is the one suggested by several authors in recent years, which uses the ORE as a measure of the tendency that individuals have to think categorically about outgroup racial members, leading them to process facial features differently from own-race faces^13,14^. Whereas ingroup faces (e.g., own-race) facilitate the perceivers to search for facial features that could distinguish one ingroup member from another, outgroup faces (e.g., other-race) are categorised based on category-prototypical features (e.g., race, sex, age) that are common across all the outgroup faces, thus making discrimination more difficult. Essentially, the argument is that there are different facial features used within own and other-race faces that are guided by social categorisation based on group membership, in this case race, but the same analysis could apply to sex and age^15^.

Cognitive scientists have instead proposed an explanation of the ORE based on the lack of visual experience we have with other-race individuals which would result in reduced perceptual expertise with other-race faces. Researchers provided evidence that the size of the ORE would vary with the amount of interracial contact experience that individuals have in their everyday life^16,17^. Interestingly, a recent study also proposed that there would be a specific developmental window (approximately until 12 years of age) where acquisition of other-race faces is facilitated and would have effects in the reducing the ORE^18^. Furthermore, authors have explored the mechanisms at the basis of the role that perceptual expertise plays in determining the ORE. More specifically, it has been proposed that the perceivers would have more expertise at scrutinising the configural information (i.e., spatial relationships among the main facial features) for own-race faces versus other-race faces with the latter being processed more featurally (i.e., isolated features)^5,15^. It is this visual perceptual expertise for configural information that we rely on when recognising faces as demonstrated by the face inversion effect (FIE)^19,20^. Currently, 300 + papers have shown that when we are presented with upside-down faces our recognition performance is significantly reduced compared to when we see the same faces presented in their usual upright orientation. This is the FIE and it is another robust phenomenon in the face recognition literature. The most widespread explanation for the FIE is that when presented upright, we process faces based on their configural information, however, when inverted configural information is disrupted resulting in a reliance on individual features and a reduced recognition performance^21–24^.

Importantly, Rhodes et al. adopted the FIE to investigate the perceptual expertise explanation of the ORE^25^. The authors hypothesised that if own-race faces are processed more configurally than other-race faces, a reduced FIE should be recorded for other-race faces due to there being less expertise at scrutinising configural information to be lost on inversion. Western Caucasian and East Asian students were recruited and engage with a recognition task involving Western Caucasian and East Asian upright and inverted faces. As predicted the results revealed for both subject samples, a larger FIE for own-race versus other-race faces supporting the perceptual expertise linked to configural processing explanation of the ORE (for a replication see^26^). Vizioli et al.^27^ using the old/new recognition task typically adopted to investigate the FIE, showed that for Western Caucasian subjects the FIE was larger in response to Western Caucasian faces versus that found for East Asian faces mainly because of impaired recognition performance for upright East Asian Faces. Critically, this result was reversed for East Asian subjects, thus a larger FIE was found for East Asian faces versus that found for Western Caucasian faces and this was mainly because of impaired recognition performance for upright Western Caucasian faces^27^. Within the same study Vizioli et al.^27^ looked at the ORE on the N170 ERP component which is often used in the literature as an index of face recognition performance, and it is found to be the largest at occipital-temporal areas^28^. Typically, inverted faces elicit a delayed and larger N170 peak compared to that in response to upright faces^29^. The results revealed that for both Western Caucasian and East Asian subjects the FIE on the N170 amplitudes was larger for same versus other race faces revealing a similar pattern to the behavioural effects. No significant differences were found on the N170 latencies^27^.

Vizioli et al.’s behavioural results are particularly interesting in the context of the ORE literature^27^. More specifically, the reduced expertise for upright other-race faces, which leads to impaired recognition performance, is what leads to a smaller FIE compared to that found for own-race faces. On inversion, because we are not familiar with seeing either own or other-race faces presented inverted, no difference in performance should be expected. However, these results could be also explained by the social accounts of the ORE. One may say that either the lack of motivation to approach individuals from other races, or the tendency to categorise outgroup members based on prototypical elements (e.g., race) could mainly affect upright faces thus leading to a reduced FIE for other-race faces. This illustrates a key limitation in this literature thus far, there has been a persistent failure to develop an experimental paradigm that could definitively demonstrate whether the ORE is due to either social factors or cognitive mechanisms by excluding one or the other. Interestingly, recent studies have also attempted at reconciling these two extreme accounts (social motivation and perceptual expertise) proposing that increasing motivation may be helpful only if a sufficient minimum degree of perceptual experience is present, or that motivation contributes over-and-above experience only in cultural settings where the groups differ in socioeconomic status^30^. Some authors proposed that the ORE might be due to a lack of social motivation in some circumstances, and a lack of perceptual experience in others^31^. But perhaps, the most developed hybrid account is that based on the Categorization-Individuation Model^32^. This is still a socially orientated theory of the ORE which is based on subjects’ selective attention to category-level or identity-level facial features, but it proposes that experience influences the ease with which it is possible to selectively attend to identity-level information. Thus, in subjects with strong other-race experience, attending to identity-level information will be easy and successful while in subjects with weak other-race experience, attending to identity-level information, even when motivated to do so, could be difficult or unsuccessful. A few studies have shown that if subjects are motivated to apply the same individuation processes (e.g., informing them of the presence of other-race faces and encouraging them not to avoid them) then if they are sufficiently familiar with the race (e.g., Western Caucasian Americans looking at African American) the ORE can be overcome^32,33^.

In the current study we aimed to examine further the nature of the ORE directly by removing the perceptual expertise component for own-race faces and seeing whether that would eliminate the ORE. To do so, we used a transcranial Direct Current Stimulation (tDCS) procedure developed in the literature in recent years, to directly affect and disrupt the perceptual learning component of the FIE. The specific tDCS montage targeting the DLPFC area with anodal stimulation derives from previous research on categorisation learning tasks^34–36^. Specifically, Ambrus et al.^36^ provided the first evidence for anodal tDCS delivered over the left DLPFC at Fp3 area influencing categorization learning for sets of prototype-defined pattern configurations. The DLPFC region was chosen based on previous fMRI studies showing this brain region being highly activated throughout the whole learning phase of the categorisation task used^37^. The Fp3 area was selected because of being particularly implicated in participants with high categorization performance^36,37^. In Ambrus et al.^36^ the authors administered the tDCS stimulation for a brief period of 10 min during the learning phase (i.e., online) and made sure that stimulation ended before the beginning of the testing phase. The results revealed that anodal tDCS influenced categorisation in the testing phase directly eliminating the prototype distortion effect (better categorization performance for non-pre-exposed category prototypes compared to category exemplars). This finding was later extended by other authors^38^ to a prototype-distortion task using the same prototype-defined categories of checkerboards used in the literature to demonstrate an analogous of the FIE^39–41^. McLaren et al.^38^ showed that anodal tDCS at Fp3 (cathode/return channel placed on the opposite supraorbital area) delivered for 10 min at an intensity of 1.5 mA, during the learning phase reduced later in the testing phase the prototype distortion effect for checkerboards.

Through, a double-blind and between-subjects design Civile, Verbruggen et al.^42^ extended the same tDCS procedure to modulate the robust inversion effect with checkerboards previously established in the literature as evidence of perceptual learning^39–41^. Anodal stimulation delivered during the categorization learning task (at Fp3 area, 10 min duration, 1.5 mA intensity) involving prototype-defined categories of checkerboards influenced the checkerboard inversion effect recorded in the subsequent old/new recognition task. Specifically, the checkerboard inversion effect in the anodal group was significantly reduced compared to the robust checkerboard inversion effect found in the sham group. Importantly, the reduction of the inversion effect was mainly due to impaired performance for upright familiar checkerboards in the anodal condition versus sham^42^. Critically, when the same tDCS procedure was extended to the inversion effect for faces a similar pattern of results was demonstrated. Civile et al.^43^ used the same tDCS procedure while participants were engaged in an old/new recognition task involving Western Caucasian upright and inverted faces (Experiment 1 and 2). A double-blind and between-subjects design was adopted where the tDCS stimulation was delivered throughout the learning phase (study phase). The results from the subsequent recognition task revealed a robust FIE in the sham group and a significantly reduced FIE in the anodal group. This time as well this reduction was due to an impaired performance for upright faces induced by the anodal tDCS^43^. This effect of the tDCS procedure on the FIE has been replicated across several publications and constitutes an established finding in the literature^43–49^. This work established a causal link between the inversion effect for faces and that for checkerboards, by showing that tDCS can systematically reduce both.

There are two key aspects regarding in the development of the tDCS procedure used to modulate the inversion effect. Firstly, Civile et al.^43^ conducted an active control study (Experiment 3) to investigate whether similar effects on the FIE would be obtained when another area was targeted. The authors selected the rIFG area (cathode/return channel placed on the opposite supraorbital area, Fp1) based on previous studies that have shown anodal tDCS delivered on this area to be effective at modulating several tasks (e.g., go/no go tasks^50,51^) however it had never been investigated in response to perceptual learning tasks. Civile et al.^43^’s Experiment 3 found no differences between the robust FIE found in the sham group versus that found in the anodal tDCS at rIFG group. Secondly, in a recently published paper Civile et al.^49^ directly compared the effects of anodal tDCS at Fp3 versus anodal tDCS at PO8 while subjects performed a composite face effect study involving upright face only. Subjects were randomly assigned to three tDCS groups including anodal tDCS at Fp3, anodal tDCS at PO8 and sham (split by the two montages). In all three groups the cathode/return channel was placed on the right supraorbital (Fp2) in line with previous studies that used the tDCS at Fp3 procedure on the inversion effect. The specific PO8 site was selected based on EEG literature on the N170 ERP component that revealed how the FIE is found to be largest on that channel^28,29^. Moreover, a few previous tDCS studies had found different pattern of results on face recognition tasks when PO8 and closely related areas had been stimulated. Civile et al.^49^’s findings revealed no effect of tDCS on the size of the composite face effect (which is better recognition of the top half of an upright face when conjoined with a congruent rather than an incongruent bottom half) however, it confirmed the reduction in overall recognition performance (the task involved all upright faces) for subjects in the anodal tDCS at Fp3 group versus the sham group and versus also the tDCS at PO8 group. Critically, no differences were found between the sham and the anodal tDCS at PO8 groups. Thus, these results would confirm that the tDCS-induced effects on perceptual learning for upright faces would seem to be related to anodal tDCS at the Fp3 area, and when that is moved to PO8 accompanied by leaving the cathode/return channel location in the same location no effects are obtained. These results also extended this new line of research looking at the effects of tDCS delivered at occipital sites on face recognition tasks. The investigation is ongoing with studies that have demonstrated different effects depending on the task and the experimental design used. For instance, Barbieri et al.^52^ showed how a single-blind offline (pre- behavioural task) anodal tDCS at PO8 (20 min duration, 1.5 mA intensity) can induce higher face and object recognition performance. Yang et al.^53^ showed how a single-blind online anodal tDCS at P8 (15 min duration, 1.5 mA intensity) can influence face recognition skills indexed by the size of the composite face effect, however, no further analyses were provided to establish if the effects were due to an enhanced or reduced performance for any of the specific conditions. Renzi et al.^54^ using a single-blind, within-subjects design targeting a closely related area (OFA area), found that anodal tDCS (20 min duration at 2 mA intensity) did not influence the composite face effect (similarly to Civile et al.^49^). However, when the authors applied the same tDCS procedure to a Mooney Task (black and white distorted images) a blocking learning effect was found at face detection and a decreased performance at object detection^54^.

In our current study we aimed to investigate directly how tDCS can modulate the ORE. We used the specific tDCS procedure (anodal tDCS at Fp3, for 10 min, at 1.5 mA) developed in the perceptual learning and face recognition literature as a tool to directly affect the robust checkerboard and face inversion effects both indices of perceptual learning. The reasoning here is that, if in typical circumstances a component of the ORE is the reduced perceptual expertise for other-race faces, the tDCS procedure should alter this by disrupting the perceptual expertise component, manifesting through perceptual learning, for own-race faces (i.e., they are more familiar). Little or no effect of the tDCS procedure should be expected for other-race faces considering that there is less expertise to be lost for these faces. The tDCS procedure should selectively reduce the FIE for own-race faces and as a consequence, this would cause a reduction of the overall cross-race interaction (FIE for own vs other-race faces) that is used as an index of the ORE. Contrarily, if we assume that individuals have visual expertise for both own and other-race faces (they are all faces) and the ORE is specifically based on a lack of social motivation to approach individuals from another race, or social categorisation, the tDCS procedure would reduce the FIE for both own and other-race faces, and the ORE would still be found to be significant i.e., larger FIE for own versus other race faces.

## Methods

### Subjects

Overall, 96 naïve self-declared Western Caucasian subjects (62 women; mean age = 20.8, age range = 18–34 years) took part in the study. The subjects were randomly assigned to either sham or anodal tDCS groups (48 in each group). All the subjects were University of Exeter students who have lived in Exeter (a town in the south-west of England with around 90% Western Caucasian population) for at least two years and before that they all lived in countries where Western Caucasian faces are largely predominant (United Kingdom, Germany, Italy, Spain, Poland, France, Bulgaria Romania, Canada, USA). Subjects were selected according to the tDCS safety screening criteria. All methods were performed in accordance with the relevant guidelines and regulations approved by the College of Life and Environmental Sciences, Psychology Research Ethics Committee at the University of Exeter. Informed consent was obtained from all subjects.

The sample size was established based on previous studies that have used the same tDCS procedure, behavioural paradigm, and counterbalance of the stimuli, to investigate the effects of tDCS on the FIE^43–49^.

### Materials

The study used a set of high resolution 80 Western Caucasian and 80 East Asian male and female faces (5.63 cm × 7.84 cm) standardized to grayscale on a white background. These images were selected from the open access Chicago Face Database: Multiracial Expansion^55^. This database was created for research with a focus on the perception and racial categorization of multiracial individuals. It includes a free set of high-resolution, standardized images featuring real multiracial individuals along with extensive norming data and objective physical measures of these faces. These data are offered as an extension of the widely used Chicago Face Database and are available for download at www.chicagofaces.org for use in research. This face database has been used often in the literature to examine the ORE across different tasks^56–60^.

The experiment was run using Superlab 4.0.7b. on an iMac computer. Participants sat about 70 cm away from the screen on which the images were presented.

### tDCS apparatus

**Figure 1:**
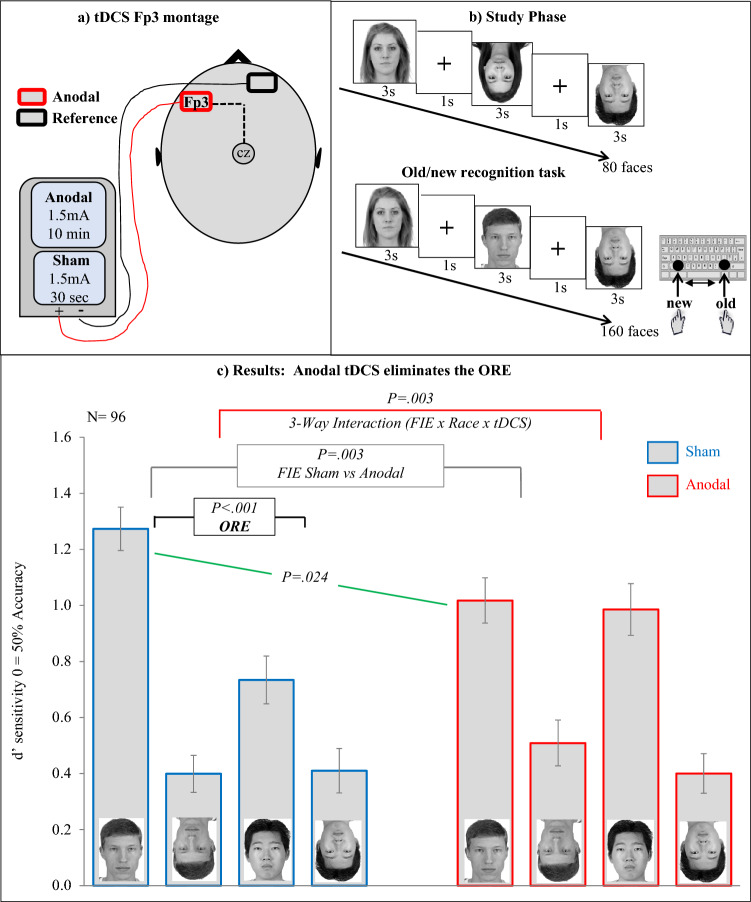
(**a**) The tDCS montage adopted in the study. (**b**) The *old/new recognition* task used. (**c**) The results from the study. The *x*-axis shows the face types in each tDCS group. The *y*-axis shows sensitivity *d*′ measure. Error bars represent s.e.m. The face images were selected from the open access Chicago Face Database (www.chicagofaces.org)^55–60^. Publishing permission of these images was granted from the Center for Decision Research.

The stimulation was delivered by a battery driven constant current stimulator (neuroConn DC-Stimulator Plus) using a pair of surface sponge electrodes (7 cm × 5 cm i.e., 35 cm^2^) soaked in saline solution and applied to the scalp at the target areas of stimulation. In agreement with previous work^43–49^ we adopted a bilateral bipolar-non-balanced montage with one of the electrodes (anode) placed over the target stimulation area (Fp3) and the other (cathode/return) on the opposite supraorbital area (above the right eyebrow). Using a tDCS EEG-cap based system (e.g., Starstim System) the location of the cathode/return electrode would correspond to the Fp2 channel (for an example see Civile et al.^46^). We used a double-blind procedure reliant on the neuroConn study mode in which the experimenter inputs numerical codes (provided by another experimenter otherwise unconnected with running the experiment), that switch the stimulation mode between “active” (i.e., anodal) and “sham” stimulation. In the anodal condition, a direct current stimulation of 1.5 mA was delivered for 10 min (5 s fade-in and 5 s fade-out) starting as soon as the subjects began the learning phase (study phase) which lasted for approximately 5 min, and continued into the recognition task which lasted approximately 10 min. In the sham group, subjects experienced the same 5 s fade-in and 5 s fade-out, but with the stimulation intensity of 1.5 mA delivered for just 30 s, following which a small current pulse (3 ms peak) was delivered every 550 ms (0.1 mA over 15 ms) for the remainder of the 10 min to check impedance levels (see Fig. 1a).

### Behavioral task

The old/new recognition task consisted of two parts: a ‘study phase’ and an ‘old/new recognition phase’^43–47^. In the study phase, each subject was shown 40 upright (20 male and 20 female) and 40 inverted (20 male and 20 female) Western Caucasian and East Asian faces. The faces were shown one at a time in random order with no response required from the subject. In the old/new recognition phase, 80 novel faces (half upright and half inverted) were added to the 80 faces seen during the study phase. All 160 faces were presented one at a time in random order and the subject had to respond according to whether they thought they had seen the faces during the study phase. For a given subject, each face stimulus only appeared in one orientation during the experiment.

Following the instructions, in each trial of the study phase subjects saw a fixation cross in the centre of the screen, presented for 1 s, then a face stimulus was presented on screen for 3 s before moving on to the next trial. After all the 80 faces had been presented, the program displayed another set of instructions, explaining the recognition task. In this task, subjects were asked to press the ‘.’ key if they recognized the face stimulus as having been shown in the study phase on any given trial, or press ‘x’ if they did not (the keys were counterbalanced). During the recognition task, the face stimuli were each shown for 3 s (and stayed on the screen for whole duration) during which time subjects had to respond (see Fig. 1b).

## Results

### Data analysis

Our primary measure was performance accuracy in the old/new recognition task. As in previous studies^43–49^ the data from all the subjects in each experimental condition was used to compute a *d*-prime (*d*′) sensitivity measure^61^ for the recognition task where a *d* = of 0 indicates chance-level performance. We assessed performance against chance to show that both upright and inverted Western Caucasian and East Asian faces in both the tDCS sham and anodal groups were recognized significantly above chance. (For all four conditions we found *p* < .001 for this analysis). For completeness we also analysed the data for decision criterion, *C*, which in agreement with previous studies^43–49^ revealed no effects of the tDCS procedure on *C*. We do not report the *C* analyses because they do not add anything to the interpretation of the results. Each p-value reported for the comparisons between conditions is two-tailed, and we also report the F or t value along with effect size ($\upeta _{{\text{p}}}^{2}$).

We computed a 2 × 2 × 2 mixed model design using, as a within-subjects factor, *FIE* (upright or inverted), and *Face Race* (Western Caucasian or East Asian) and the between-subjects factors *tDCS Stimulation* (sham or anodal). A mixed model Analysis of Variance (ANOVA) revealed a significant main effect of *FIE, F*(1,94) = 155.83, *p* < .001, $\upeta _{{\text{p}}}^{2}$ = .62 and *Face Race*, *F*(1,94) = 11.73, *p* < .001, $\upeta _{{\text{p}}}^{2}$ = .11. No significant main effect of *tDCS Stimulation* was found, *F*(1,94) = .10, *p* = .75, $\upeta _{{\text{p}}}^{2}$ < .01. No significant interaction was found for *FIE* × *tDCS Stimulation*, *F*(1,94) = .32, *p* = .57, $\upeta _{{\text{p}}}^{2}$ < .01. A significant interaction was found for *FIE* × *Face Race*, *F*(1,94) = 5.41, *p* = .022, $\upeta _{{\text{p}}}^{2}$ = .05, and for *Face Race* × *tDCS*, *F*(1,94) = 3.93, *p* = .050, $\upeta _{{\text{p}}}^{2}$ = .04. Critically, the overall three-way interaction, *FIE* × *Face race* × *tDCS Stimulation* was significant, *F*(1,94) = 9.47, *p* = .003, $\upeta _{{\text{p}}}^{2}$ = .09. We decomposed this overall interaction by examining the two-way interactions (*FIE* × *Face Race*) separately for each tDCS condition.

#### Sham tDCS group

A 2 × 2 ANOVA revealed a significant main effect of *Face Race, F*(1,47) = 16.67, *p* < .001, $\upeta _{{\text{p}}}^{2}$ = .26, and *FIE, F*(1,47) = 89.89, *p* < .001, $\upeta _{{\text{p}}}^{2}$ = .65. Importantly, a significant interaction was found, *F*(1,47) = 13.21, *p* < .001, $\upeta _{{\text{p}}}^{2}$ = .21. Paired-sample t-tests showed a significant inversion effect was found for Western Caucasian faces (M = .87, SD = .64), *t*(47) = 9.35, *p* < .001, $\upeta _{{\text{p}}}^{2}$ = .65, and, critically, a *reduced* inversion effect for East Asian faces (M = .32, SD = .71), *t*(47) = 3.13, *p* = .003, $\upeta _{{\text{p}}}^{2}$ = .37, essentially confirming a robust ORE (see Fig. 1c). An additional analysis showed that recognition for upright Western Caucasian faces was significantly better than that for upright East Asian faces, *t*(47) = 5.36, *p* < .001, $\upeta _{{\text{p}}}^{2}$ = .38. No difference was found for inverted Western Caucasian faces versus inverted East Asian faces, *t*(47) = .11, *p* = .91, $\upeta _{{\text{p}}}^{2}$ < .01 (see Fig. 1c).

#### Anodal tDCS group

A 2 × 2 ANOVA revealed no significant main effect of *Face Race, F*(1,47) = .92, *p* = .34, $\upeta _{{\text{p}}}^{2}$ < .01, and a significant main effect of *FIE, F*(1,47) = 67.43, *p* < .001, $\upeta _{{\text{p}}}^{2}$ = .58. No significant interaction was found, *F*(1,47) = .31, *p* = .58, $\upeta _{{\text{p}}}^{2}$ < .01 indicating that the FIE for own-race faces was no longer significantly larger than the FIE for other-race faces (see Fig. 1c).

#### Additional analysis between tDCS groups

We first calculated the FIE index (performance for upright faces – that for inverted faces) for Western Caucasian faces in each tDCS group. Then, we conducted an independent sample t-test which showed that the inversion effect for Western Caucasian faces in the anodal group was significantly reduced compared to that in the sham group, *t*(94) = 3.02, *p* = .003, $\upeta _{{\text{p}}}^{2}$ = .08. Critically, performance for upright Western Caucasian faces in the anodal group was also significantly reduced compared to that in the sham group, *t*(94) = 2.28, *p* = .024, $\upeta _{{\text{p}}}^{2}$ = .05. No significant difference was found between inverted Western Caucasian faces in the anodal versus sham groups, *t*(94) = 1.04, *p* = .30, $\upeta _{{\text{p}}}^{2}$ = .01 (see Fig. 1c). The difference between the inversion effect indices for East Asian faces in the anodal versus sham group was not significant, *t*(94) = 1.72, *p* = .087, $\upeta _{{\text{p}}}^{2}$ = .03.

## Bayes factor analyses

Using the procedure outlined by Dienes^62^ we first conducted a Bayes analysis on the difference between the robust ORE found in the sham group versus the eliminated ORE in the anodal tDCS group (thus capturing the significant 3-Way interaction). Given that the effect (i.e., ORE) can be as large as is found in the sham condition, is the effect (i.e., the reduced ORE) found in the anodal condition part of that population, or is it better described as null (mean of zero)?

We used as the *prior* the two-way interaction (*Face Race* × *tDCS Stimulation*) index of the ORE setting the standard deviation of p (*population value | theory*) to the mean for the difference between the FIE score (upright – inverted) for Western Caucasian faces versus the FIE score for East Asian faces in the sham group [0.55]. We used the *standard error* [0.13] and *mean difference* [− 0.08] between the FIE score for Western Caucasian faces versus the FIE score for East Asian faces in the anodal group. We assumed a one-tailed distribution for our theory and a mean of 0. This gave us a Bayes factor of 0.14 which is strong evidence in support for the null (less than 0.30 for the conventional cut-offs see^62,63^), supporting the claim that the anodal stimulation procedure eliminates the ORE.

We conducted a further Bayes analysis on the inversion effect score for Western Caucasian faces comparing the sham and anodal groups (thus capturing the 2 × 2 interaction). We used as the *priors* the differences found in Civile et al.^43^ (Experiment 1 and 2 averaged together) setting the standard deviation of p (*population value | theory*) to the mean for the difference between the inversion effect in sham group versus that in the anodal group [0.30]. We used the *standard error* [0.09] and *mean difference* [0.36] between the inversion effect for Western Caucasian faces in the sham group versus that in the anodal group. We assumed a one-tailed distribution for our theory and a mean of 0. This gave a Bayes factor of 882.80, which is very strong evidence (greater than 10^62,63^) that these results demonstrate how the tDCS procedure used here reduces the face inversion effect for Western Caucasian faces.

It could be argued, however, that whilst this convincingly establishes that the effect seen under anodal stimulation (the reduced FIE) is different from the effect seen from the sham stimulation (a robust FIE), this analysis does not test directly whether the effect obtained (smaller FIE for Western Caucasian faces in the anodal vs sham group) is line with our previous studies that have shown this effect^43^. Hence, to assess this possibility, we conducted two additional analyses where the normal distribution was centred around the *prior* mean indexed by the difference between the FIE in sham group versus that in the anodal group [0.30] found in Civile et al.^43^. To do that, we first subtracted the *prior* mean [0.30] from the *mean difference* [0.36] between the inversion effect for Western Caucasian faces in the sham group versus that in the anodal group. Hence, our *mean of sample* was 0.06 and the *standard error* was still 0.09. In the analysis just performed, the *prior* mean was used as both the standard deviation and mean of p (*population value | theory*). This time we used a two-tailed distribution for our theory. This gave a Bayes Factor of 0.26 supporting that the effects are in line with the theory. In the second analysis, we only changed the mean of the theory [to − 0.30] so as to reflect the idea that if there was no effect that’s what it would be. All the other values stayed the same as for the first analysis. This gave a Bayes Factor of 0.18. These Bayes factors support the null in these analyses, but the null is now adjusted to be the mean difference expected on the basis of our previous work. Thus, we have good evidence that it is plausible to assume that our current difference is drawn from the distribution that produced our previous results.

Finally, we also conducted a Bayes factor analysis using as priors the mean difference between sham upright faces and anodal upright faces found in Civile et al.’s^43^ Experiment 1 and 2 averaged together [0.28]. We then used the standard error [0.08] and mean difference [0.25] between sham upright faces and anodal upright faces for Western Caucasian faces. This gave a Bayes factor of 50.18, which is also very strong evidence for the position that performance to upright Western Caucasian faces is reduced by the tDCS procedure, consistent with previous results.

## Discussion

The current study aimed to investigate the nature of the ORE. Using a tDCS procedure devised to remove the perceptual learning component of the FIE^43–49^ we demonstrated that the ORE can be eliminated compared to the robust ORE found in the sham/control group. Our results show that once the FIE for own-race faces has been significantly reduced by anodal tDCS (compared to sham) then the cross-race interaction used as an index of the ORE is no longer significant. Importantly, the anodal tDCS did not reduce the FIE for other-race faces supporting the hypothesis that there is less perceptual learning to be lost for those faces. Finally, we found that recognition performance for upright own-race faces, was significantly higher than for other races faces in the sham condition, but significantly reduced in the anodal condition. Furthermore, our Bayesian analyses provided support for the reduction of the ORE in the anodal group. Also, they confirmed how the reduction of the FIE for own-race faces in the anodal versus sham group and the reduced performance for upright own-race faces in the anodal group versus group are in line with previous results in literature^43–49^.

In line with previous studies that have used the same tDCS procedure applied to the FIE, our explanation for the reduced FIE for own-race faces is based on the McLaren, Kaye, and Mackintosh (MKM) theory of perceptual learning^64–66^. According to this theory, in normal circumstances experience with a prototype-defined category of stimuli (e.g., Western Caucasian faces) leads to perceptual learning. This improves discrimination between upright faces taken from this category. Hence, when observers are first exposed to the category exemplars they would focus on the prototypical/common features shared by all the exemplars. This allows them to correctly associate the exemplars with the correct category membership (e.g., Biden’s face is Western Caucasian). Once that the common features are strongly associated with category membership, they would tend to be slower at making new associations, because they would lose their salience leaving the unique features in each exemplar highly salient. This feature salience modulation process leads to perceptual learning, because now observers can focus on the unique features of each exemplar. They can now better discriminate exemplars within the same category (e.g., Biden vs Trump’s face) and recognise them easily when presented in their usual upright orientation. On inversion this benefit of perceptual learning would be lost, because we are not as familiar with upside down faces. When the tDCS procedure is applied, feature salience modulation is altered such that, the common features maintain their salience relatively high thus making the commonalities amongst faces more prominent rather than exaggerating the differences essentially making the faces to look more “similar”. It is this change in perceptual learning that causes the reduction in the FIE because it reduces the ability to discriminate between different upright faces which is normally enhanced by expertise for face processing acquired via experience^43–49^.

Given our results, there can be little doubt that perceptual expertise, manifesting through perceptual learning, for upright faces taken from a familiar (i.e., own-race) category contributes to the ORE. The may also be a hint that the explanation is not quite as simple as saying that once this component of the FIE is eliminated for own-race faces the effect is lost. We note that whilst the stimulation by orientation interaction for other-race faces is not significant, it is also not far off, and that there is, at least numerically, an improvement in performance to upright other-race faces as a consequence of anodal tDCS. It may be premature to speculate on this point in the absence of definitive statistical support, but our results are somewhat reminiscent here of effects obtained in similar circumstances when studying Western Caucasian ‘regular’ faces intermixed with Thatcherized versions (the eyes and mouth have been rotated by 180°) of the same type of face^46,67^. Authors have shown that the same tDCS procedure used in our current study, can remove the negative effects of generalization induced by Thatcherized faces onto regular faces when presented intermixed within the same old/new recognition task. It was found that the anodal tDCS in this circumstance increased the FIE for regular faces compared to the reduced FIE found in the sham group (due to the negative generalization brought by Thatcherized faces), and performance for upright regular faces was significantly higher in the anodal group versus sham^45^. Coming back to results from our current study, one may argue that part of the reason for weaker performance to upright other-race faces in the sham group is generalization from own-race faces, and this effect is reduced by tDCS allowing performance for other-race faces to recover. This would rely on the distinctive, salient features in own-race faces being the ones that generalize to other-race faces, as this would both be diminished by tDCS and provide an asymmetric effect (i.e., no significant generalization in the reverse direction). Future work should examine this further by comparing the tDCS-induced effects on recognition performance for other-race faces when presented intermixed with own-race faces (i.e., stimuli that generalize onto the other-race faces) versus when other-race faces are intermixed with stimuli that do not generalize onto them (e.g., checkerboards).

A final note regarding our results is that no effects of the tDCS procedure were found on decision criterion *C*. This is in line with previous studies that have used the same tDCS procedure applied on the FIE using an old/new recognition task or a matching task^43–49^. The effects of the tDCS procedure were always found on recognition accuracy (reaction times were analyzed to check for speed-accuracy trade-off and none was found), and d-prime was used as a measure of discriminability. However, several authors have suggested how typical recognition tasks such as an old/new recognition would preclude a detailed investigation of response criterion effects because it would tend to lead to a "balanced" effect on *C* that cancels any potential effect out^68,69^. To our knowledge, only one recently published study found that the tDCS can modulate criterion (but not discriminability) on a perceptual learning and face recognition task involving faces and checkerboards. However, the authors used a quite different and specifically designed *target detection task* of the kind previously used in the literature to study *C* although perceptual learning and applications of tDCS had never been applied to this paradigm before^70^.

Our findings contribute directly to the ORE literature by showing how a specific tDCS procedure developed in the perceptual learning literature can modulate the FIE for own-race faces leading to a full reduction of the ORE. This provides additional support to the perceptual expertise explanation of the ORE and specifically to the perceptual learning account of the FIE. Our findings do not preclude the possibility for other factors (e.g., racial bias or social motivation) to contribute to the several robust effects found in the literature regarding the perception and categorisation of multiracial individuals^56–60,71^. However, regarding the specific nature of the ORE indexed by the FIE, our findings would suggest that perceptual expertise can fully explain the differences in the size of the FIE found previously for own versus other-race faces^25–27^. Importantly, whereas there are several studies having shown how the tDCS procedure used here influences perceptual learning^43–49^, no studies have yet reported that the same procedure could potentially influence social motivation. Thus, it is not plausible at the current moment to formulate an alternative explanation for the results obtained on the ORE indexed by the FIE, other than the one based on the perceptual expertise account. Future work should expand our study to the full cross-race design used by Vizioli et al.^27^ where both Western Caucasian and East Asian participants were recruited. A reduced ORE by means of the tDCS procedure would be expected in both groups of participants, and this would also provide the data needed to explore the more speculative analysis outlined above regarding the effects of tDCS on upright other-race faces.

More generaly, our findings contribute to the emerging literature looking at the effects of tDCS on the ORE. To our knowledge only one study^72^ before has looked, albeit indirectly, at the effects of tDCS on the ORE. Authors aimed to investigate the effects of single-blind offline cathodal tDCS at PO8 occipital area (the anode/return channel was placed on the opposite supraorbital area at Fp1 channel) on various recognition tasks that involved Western Caucasian faces and objects (the FIE was not tested). No effects of cathodal tDCS versus sham were found, however through a secondary statistical analysis the authors found that cathodal tDCS reduced face recognition performance in non-Western Caucasian subjects grouped together compared to sham tDCS. Hence, it was suggested that cathodal tDCS at occipital areas would induce ORE-like effects^72^. Despite the different tDCS procedure, study design, behavioural task, and ORE measure adopted, both studies provide a first step towards the investigation of the mechanisms at the basis of the ORE using tDCS. Our findings also contribute to the perceptual learning literature by providing further evidence in support of a tDCS procedure that can be used to systematically affect the expertise component of a specific phenomenon taken under investigation. Finally, our findings also add to the current literature regarding applications of tDCS to modulate face recognition performance using different paradigms and tasks^52–54^.

In conclusion the fact that the same tDCS procedure used in previous research to disrupt perceptual learning for checkerboard and face stimuli eliminates the ORE suggests that expertise, manifesting through perceptual learning, is the key mechanism at the basis of the ORE indexed by the FIE.

## Data Availability

The datasets generated during the current study are not currently publicly available as a precaution so that other people will not use them to produce new publications. However, these datasets are available from the corresponding author upon reasonable request.
